# Estimation of total collagen volume: a T1 mapping versus histological comparison study in healthy Landrace pigs

**DOI:** 10.1007/s10554-020-01881-x

**Published:** 2020-05-14

**Authors:** A. Faragli, S. Merz, F. P. Lo Muzio, P. Doeblin, R. Tanacli, C. Kolp, D. Abawi, J. Ötvös, C. Stehning, B. Schnackenburg, B. Pieske, H. Post, R. Klopfleisch, A. Alogna, S. Kelle

**Affiliations:** 1 Department of Internal Medicine and Cardiology, Charité – Universitätsmedizin Berlin, Campus Virchow-Klinikum, Augustenburgerplatz 1, 13353 Berlin, Germany; 2 Berlin Institute of Health (BIH), Berlin, Germany; 3 DZHK (German Centre for Cardiovascular Research), Partner Site, Berlin, Germany; 4 Institut für Tierpathologie, Freie Universität Berlin, Robert-von-Ostertag-Straße 15, 14163 Berlin, Germany; 5 Department of Surgery, Dentistry, Paediatrics and Gynaecology, University of Verona, Via S. Francesco 22, 37129 Verona, Italy; 6 Department of Medicine and Surgery, University of Parma, Via Gramsci 14, 43126 Parma, Italy; 7 Department of Internal Medicine/Cardiology, Deutsches Herzzentrum Berlin, Augustenburger Platz 1, 13353 Berlin, Germany; 8 Clinical Science, Philips Healthcare, Röntgenstr. 24, 22335 Hamburg, Germany; 9 Department of Cardiology, Contilia Heart and Vessel Centre, St. Marien-Hospital Mülheim, 45468 Mülheim, Germany

**Keywords:** Cardiovascular magnetic resonance, T1 mapping, Histology, Total collagen volume

## Abstract

Right ventricular biopsy represents the gold standard for the assessment of myocardial fibrosis and collagen content. This invasive technique, however, is accompanied by perioperative complications and poor reproducibility. Extracellular volume (ECV) measured through cardiovascular magnetic resonance (CMR) has emerged as a valid surrogate method to assess fibrosis non-invasively. Nonetheless, ECV provides an overestimation of collagen concentration since it also considers interstitial space. Our study aims to investigate the feasibility of estimating total collagen volume (TCV) through CMR by comparing it with the TCV measured at histology. Seven healthy Landrace pigs were acutely instrumented closed-chest and transported to the MRI facility for measurements. For each protocol, CMR imaging at 3T was acquired. MEDIS software was used to analyze T1 mapping and ECV for both the left ventricular myocardium (LV_myo_) and left ventricular septum (LV_septum_). ECV was then used to estimate TCV_CMR_ at LV_myo_ and LV_septum_ following previously published formulas. Tissues were prepared following an established protocol and stained with picrosirius red to analyze the TCV_histo_ in LV_myo_ and LV_septum_. TCV measured at LV_myo_ and LV_septum_ with both histology (8 ± 5 ml and 7 ± 3 ml, respectively) and T1-Mapping (9 ± 5 ml and 8 ± 6 ml, respectively) did not show any regional differences. TCV_histo_ and TCV_CMR_ showed a good level of data agreement by Bland–Altman analysis. Estimation of TCV through CMR may be a promising way to non-invasively assess myocardial collagen content and may be useful to track disease progression or treatment response.

## Background

One of the most interesting features of magnetic resonance imaging (MRI) is the sensitivity to tissue composition, reflecting the physiologic and the pathophysiologic states. The T1 relaxation time, which is defined as a measure of how fast the nuclear spin magnetization returns to its equilibrium state after a radiofrequency pulse in the MRI scanner, not only contributes to the soft tissue contrast in MRI, but is also a direct marker of the chemical environment of the individual spins [1]. This technique can be performed with or without contrast injection to amplify structural changes in the myocardium, measuring the pre-contrast or native T1 relaxation time (T1 pre) and the post-contrast T1 relaxation time (T1 post) respectively. This approach is being applied for both diagnosis and prognosis of patients with myocarditis, myocardial infarction and fibrosis in various experimental setups [1]. A previous study from Jeuthe et al. has used T1 mapping in an established model of myocarditis in rats [2]. By comparing T1 measurements with histological and immunohistochemistry findings, the group was able to show that the assessment of T1 pre and T1 post allows a precise differentiation between healthy and inflamed myocardium [2]. In another experimental study, Messroghli et al. finely demonstrated in a rat model of left ventricular (LV) hypertrophy that contrast enhanced T1 mapping can be used to detect myocardial fibrosis by measuring the myocardial extracellular volume (ECV) [3]. Furthermore, Hong et al. have demonstrated that T1 mapping, and particularly ECV measurements, can reliably and non-invasively detect early cardiotoxicity in a chemotherapy-induced cardiotoxicity rabbit model [4]. Cui et al. have validated the T1 native measurement in detecting recent myocardial infarction by a comparison with a reference histological measure of infarction size in a swine model [5].

While T1 mapping and ECV are established techniques for the evaluation of fibrosis in clinical practice [6, 7], some patients still need to undergo right ventricular (RV) biopsy for assessment of collagen content in the myocardium [8]. However, RV biopsy is an invasive technique that may be accompanied by perioperative complications and poor sample reproducibility [8, 9]. Our hypothesis is that CMR could potentially avoid or reduce RV biopsies by a more precise analysis of the collagen content. In this study, we aimed to investigate the feasibility of measuring total collagen volume (TCV) through CMR by comparing it with the TCV measured at histology.

## Methods

The experimental protocols were approved by the local bioethics committee of Berlin, Germany (G0138/17), and conform to the “European Convention for the Protection of Vertebrate Animals used for Experimental and other Scientific Purposes” (Council of Europe No 123, Strasbourg 1985).

### Experimental setup

Briefly, healthy Landrace pigs (n = 7, 54 ± 10 kg) were fasted overnight with free access to water, sedated and intubated on the day of the experiment. Anaesthesia was continued with isoflurane, fentanyl, midazolam, ketamine and pancuronium. Pigs were ventilated (Julian, Draeger, Vienna, Austria) with a FiO_2_ of 0.5, an I:E-ratio of 1:1.5, the positive end-expiratory pressure was set at 5 mmHg and the tidal volume (VT) at 10 ml kg^−1^. The respiratory rate was adjusted constantly to maintain an end-expiratory carbon dioxide partial pressure between 35 and 45 mmHg. Under fluoroscopic guidance all animals were instrumented with a Swan-Ganz catheter (Edwards Lifesciences CCO connected to Vigilance I, Edwards Lifesciences, Irvine, CA, USA). After the experiment, the pigs were sacrificed with an intracoronary 80 mmol potassium bolus. The myocardium was explanted and put in a formalin solution for fixation.

### Cardiac magnetic resonance

All CMR images were acquired in a supine position using a 3T (Achieva, Philips Healthcare, Best, The Netherlands) MRI scanner with an anterior and the built-in posterior coil element, where up to 30 coil elements were employed, depending on the respective anatomy. All the animals were scanned using an identical comprehensive imaging protocol. The study protocol included initial scouts to determine cardiac imaging planes. Cine images were acquired using an ECG-gated balanced steady state free precession (bSSFP) sequence in three LV long-axis (two-chamber, three-chamber and four-chamber) planes. The ventricular two-chamber and four-chamber planes were used to plan a stack of short-axis slices covering entire LV. The following imaging parameters were used: Repetition time (TR) = 2.9 ms, echo time (TE) = 1.45 ms, flip angle = 45°, voxel size = 1.9 × 1.9 × 8.0 mm^3^ and 40 phases per cardiac cycle.

Late gadolinium enhancement images were acquired 10 to 15 min after bolus injection of 0.2 mmol/kg gadobutrol (Gadovist; Bayer, Leverkusen, Germany) with an inversion-recovery 3-dimensional spoiled gradient echo sequence. Typical parameters were voxel size = 1.7 × 1.7 × 5 mm^3^, TR/TE = 3.3/1.6 ms, and flip angle = 15°. Inversion time to null the signal of healthy myocardium was assessed individually with the use of a Look-Locker-Sequence. Short-axis late gadolinium enhancement (LGE) views of the entire LV myocardium and 2-, 3-, and 4-chamber LGE views were obtained.

For diffuse fibrosis assessment, we acquired a single breath-hold modified Look-Locker inversion-recovery (MOLLI) sequence [10] in a basal and a mid-ventricular short-axis view, before and 10 min after contrast administration. The breath-hold was performed by reducing the tidal volume to zero during T1 mapping acquisition. An apical slice was not included to avoid partial volume effects. A 5 s(3 s)3 s scheme was employed, with inversion delays ranging from 100 ms to 5 s. Typical imaging parameters were as follows: Acquired voxel size 1.9 × 1.9 × 10 mm^3^, reconstructed voxel size 1.0 × 1.0 × 10 mm^3^, slice thickness 10 mm, TR/TE = 2.8/1.4 ms, flip angle 20°, parallel acquisition SENSE = 2, acquisition in end-diastole.

### Image analysis

**Fig. 1:**
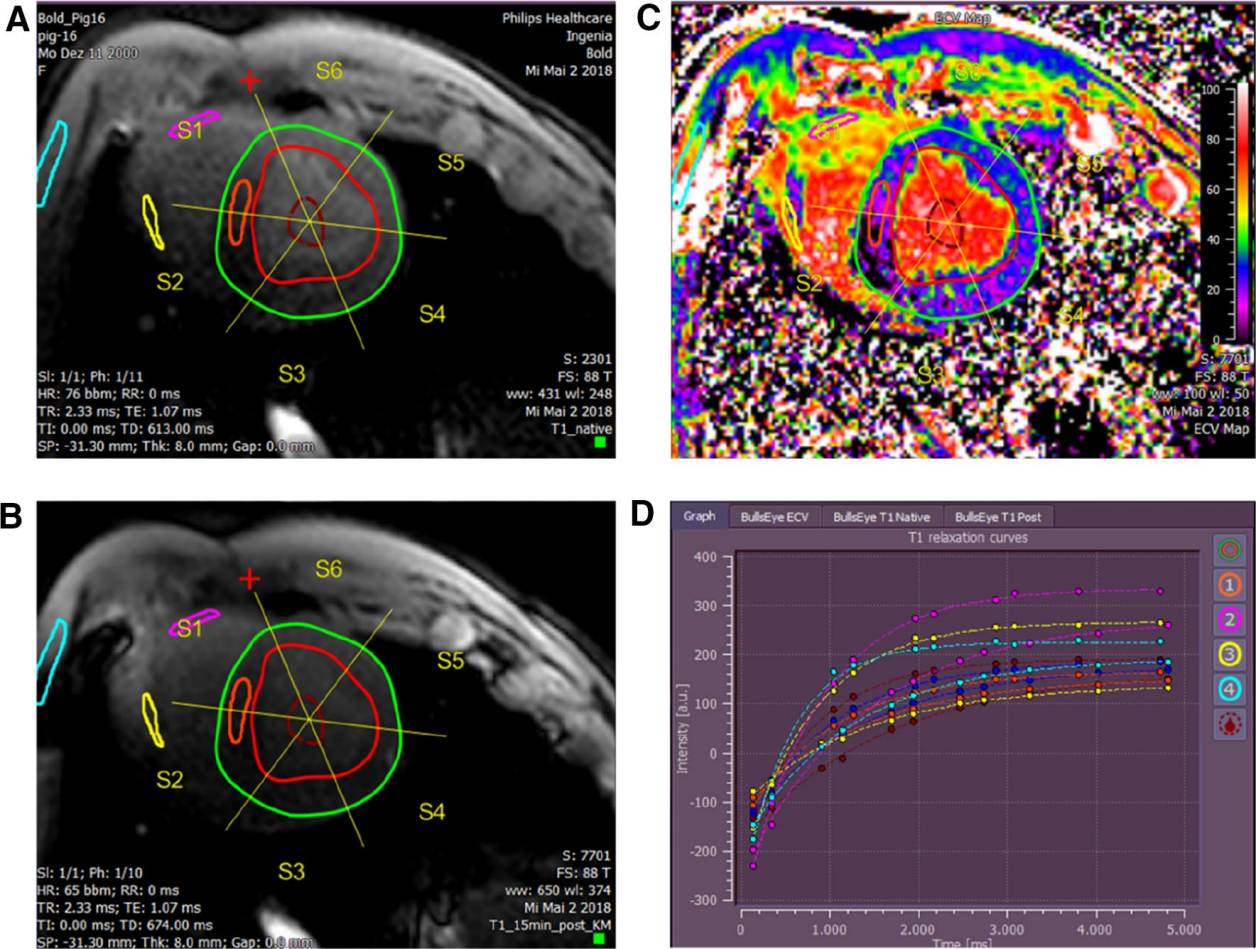
Representative T1 mapping analysis performed at medial view of one pig. **a** T1 native sequence and its related regions of interest (ROI). The ROIs are highlighted as follows: epicardial contour (green), endocardial contour (red), LV septum (orange), RV free wall (pink and yellow), blood volume (dark red) and skeletal muscle (light blue). **b** T1 post sequence after gadolinium infusion with its related ROI. **c** Postcontrast T1 quantification of the extracellullar volume (ECV). Blue color reflects high T1 values while areas depicted in green and red have lower T1 values. **d** T1 relaxation curves plotted in time for each ROI

The images obtained were analysed using a commercially available software (Medis Suite, version 3.1, Medis, Leiden, The Netherlands). Endocardial and epicardial borders were drawn in all short-axis slices at end-diastole and end-systole to calculate the global myocardial volume, myocardial mass and LV ejection fraction by using the disc summation method [11]. To calculate the T1 pre, T1 post and ECV both in the global LV myocardium (LV_myo_) and in the LV septum (LV_septum_), two different regions of interest were drawn in the myocardium and in the septum for basal and medial slices (pre-contrast and post-contrast T1 maps), with care taken to exclude the interface between myocardium and bordering structures (e.g. blood) to avoid partial volume effects (Fig. 1). A third region of interest was drawn in the blood pool.

Other regions of interest were drawn in the skeletal muscle and in the RV, but they were excluded from the analysis due to poor image resolution.

ECV was calculated as previously described [12]:

$$ECV=\left(1-hematocrit\right)\times \frac{\left(1/{T1}_{myo} post\right)-\left(1/{T1}_{myo} pre\right)}{\left(1/{T1}_{blood} post\right)-\left(1/{T1}_{blood }pre\right)}$$

where *myo* refers to the myocardial and *blood* to the blood pool T1 relaxation times, and *pre* and *post* to the measurement before and after contrast administration, respectively. For all T1 values, the averages of the basal and medial values were used.

Finally, we quantified the absolute volume of the extracellular myocardial space (absolute ECV) in each patient by using the following formula for both LV_myo_ and LV_septum_ [13]:

$$Absolute\, ECV={LV}_{myo}volume \times ECV$$

LV myocardial volume (LV_myo_ volume) represents the global LV volume expressed in millilitres and is calculated as:

$${LV}_{myo} volume=\frac{{LV}_{mass}}{1.05}$$

where 1.05 is the myocardial density given in g/ml [14].

### Total collagen volume (TCV)

The calculated ECV from the original T1 mapping of LV_myo_ and LV_septum_ was utilized to calculate the respective CVF of LV_myo_ and LV_septum_.

MRI-derived collagen volume fraction was calculated as follows [15]:

$$CVF\, \left(\%\right)=\left[\left(197\times ECV\right)\right]-36]$$

To compare CMR- and histology-derived collagen content we calculated the total collagen volume (TCV) expressed as follows:

$${TCV}_{CMR}\, \left(ml\right)= \frac{Absolute ECV \times CVF (\%)}{100}$$

### Histology

**Fig. 2:**
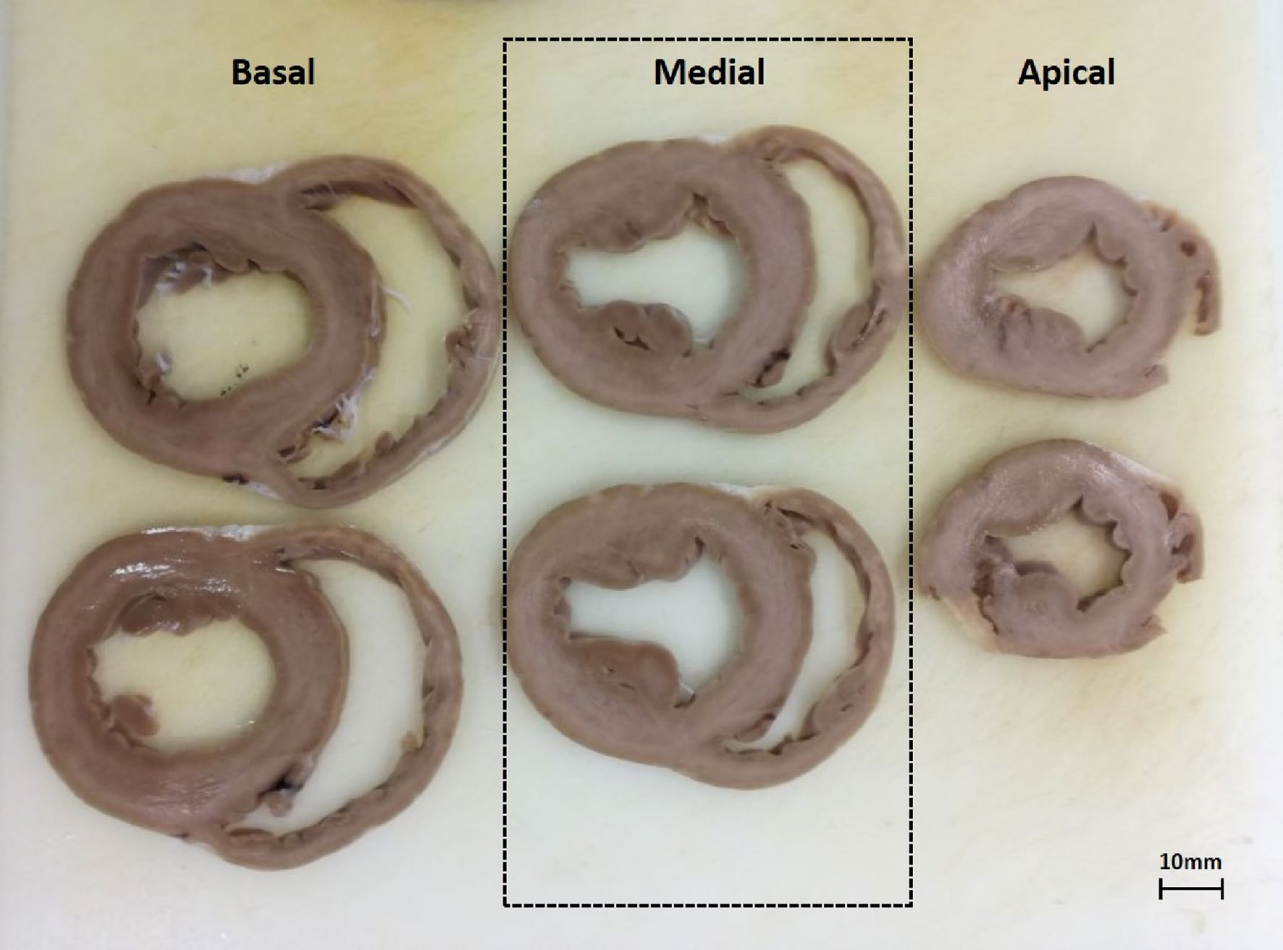
Representative picture of the histological slices. Slices obtained from the basal, medial and apical sections of a healthy pig heart. The dashed box highlights the section used for comparison with T1 mapping

**Fig. 3:**
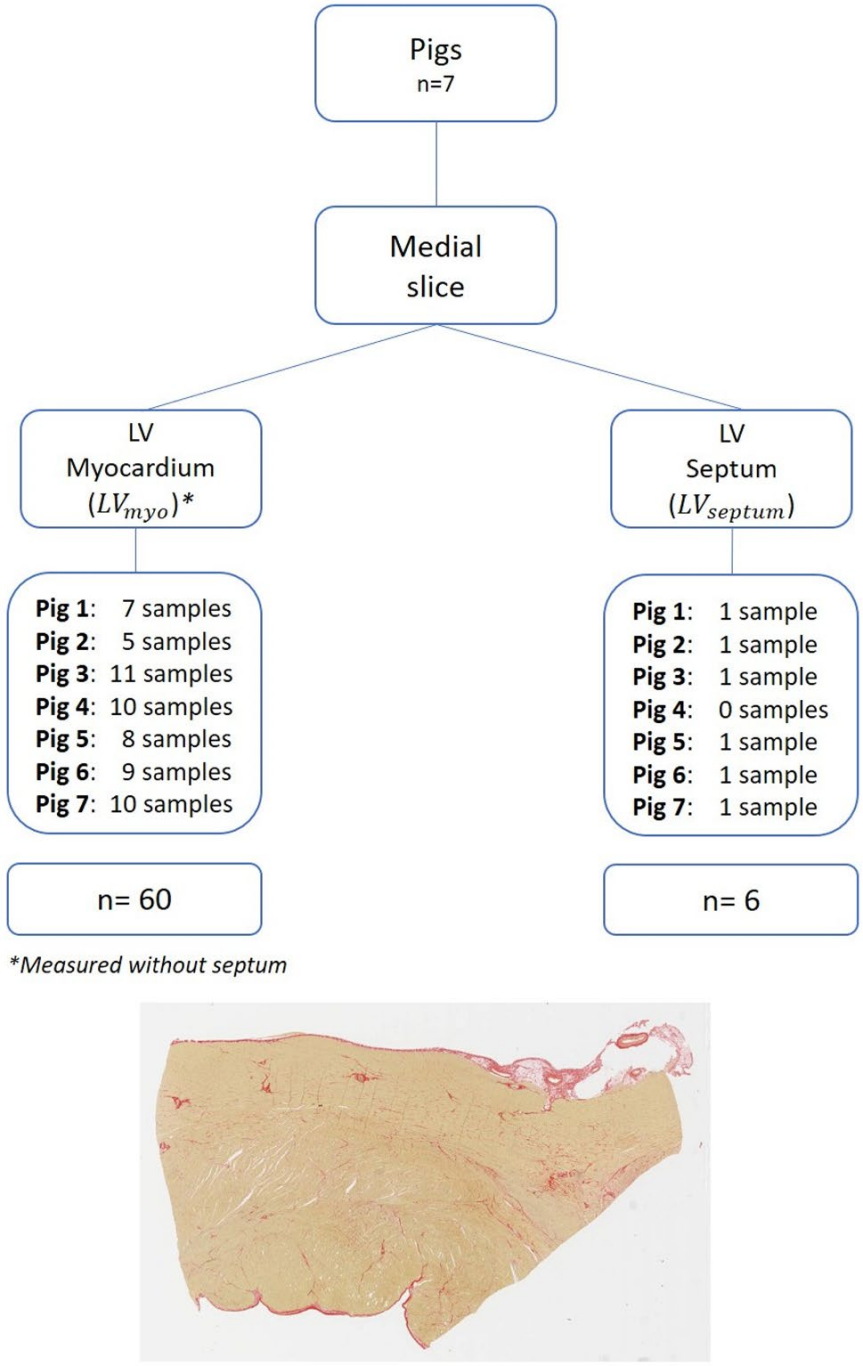
Diagram representing the histology protocol. From 7 pigs we retrieved the medial slices which were divided in left ventricular myocardium (LV_myo_) and left ventricular septum (LV_septum_) sections. For each section, the number of relative samples are displayed. The image below is a representative sample of a pikrosirius tissue staining. *LV*_*myo*_ left ventricular myocardium, *LV*_*septum*_ left ventricular septum

Whole hearts were fixed in formalin for at least 48 h and consecutively cut in approximately 1 cm thick transversal sections separating them in basal, medial and apical levels (Fig. 2). All sections that covered both ventricles were numbered and cut into smaller biopsies to fit into histology cassettes. All tissue samples were paraffin-embedded, sectioned and routinely Hematoxylin and Eosin (HE) stained. In addition, all tissue samples were picrosirius red stained according to the manufacturer’s instruction (Morphisto, Frankfurt am Main, Germany). The tissue samples were also differentiated as following: left ventricular myocardium (LV_myo_, without septum), left ventricular septum (LV_septum_) and right ventricular free wall (excluded from the analysis). HE-stained slides were analyzed qualitatively by a board-certified veterinary pathologist for morphologic abnormalities. In addition, all picrosirius red-stained slides were digitized at 20× magnification using an Aperio CS2 (Leica Microsystems Ltd, UK) slide scanner. The red-stained collagen content was determined by a software algorithm (Aperio ImageScope and Aperio GENIE, both Leica Biosystems). These whole slides images were analyzed for the quantitative proportion of collagen fibers in the tissue specimen of the different anatomic locations. The histology protocol and the number of samples used are summarized in Fig. 3. To compare the TCV measured with histology against T1 mapping with the same unit of measure we calculated as follows:

$${TCV}_{histo}\, \left(ml\right)=\frac{CVF (\%) \times { LV}_{mass}}{1.05}$$

where the collagen volume of each sample is calculated in ml, the LV mass is calculated with MRI and 1.05 represents myocardial density given in g/ml [14].

### Statistical analysis

All data are presented as mean ± standard deviation (SD). The presence of outliers was evaluated with Grubbs' test and when present they were opportunely removed. The normality of the data was assessed with the Kolmogorov–Smirnov test. Difference between groups at histology and CMR was analysed by one-way ANOVA. Paired student *t* test or Mann–Whitney test were performed, accordingly, to compare the two different techniques in the same group. The level of agreement between histology and T1 mapping was evaluated with Bland–Altman analysis. The correlations between histological parameters were assessed by linear regression analysis. A p-value < 0.05 was considered significant. The software Sigmastat (Version 4.0, Systat Software Inc., San Jose, CA) and SPSS (Version 23.0, IBM, Armonk, NY) were used for statistical analysis and results display.

## Results

### T1 mapping

All the CMR parameters analysed through T1 mapping are represented in Table 1. A significant difference was observed between T1 native and T1 post for both LV_myo_ (1232 ± 72 vs. 597 ± 334, p < 0.001) and LV_septum_ (1148 ± 70 vs. 592 ± 339, p < 0.001) respectively. A significant difference was observed between T1 native measurements between LV_myo_ and LV_septum_ (1232 ± 72 vs. 1148 ± 70, p = 0.033). No significant difference was observed between LV_myo_ and LV_septum_ either for T1 post and ECV.

**Table 1:** CMR T1 mapping parameters

Parameters	LV_myo_	LV_septum_	p-value
T1 pre (ms)	1232 ± 72	1148 ± 70	0.033
T1 post (ms)	597 ± 334	592 ± 339	0.959
ECV (%)	34 ± 5	29 ± 8	0.109
ECV (ml)	27 ± 7	23 ± 8	0.178
CVF (%)	31 ± 10	27 ± 12	0.412
TCV_CMR_ (ml)	9 ± 5	8 ± 6	0.414

Data above represent the MRI parameters measured through T1 mapping in LV_myo_ and LV_septum_

Data are presented as mean ± SD

*LV*_*myo*_ left ventricular myocardium, *LV*_*septum*_ left ventricular septum, *ECV* extracellular volume, *CVF* collagen volume fraction, *TCV* total collagen volume

### Total collagen volume

**Fig. 4:**
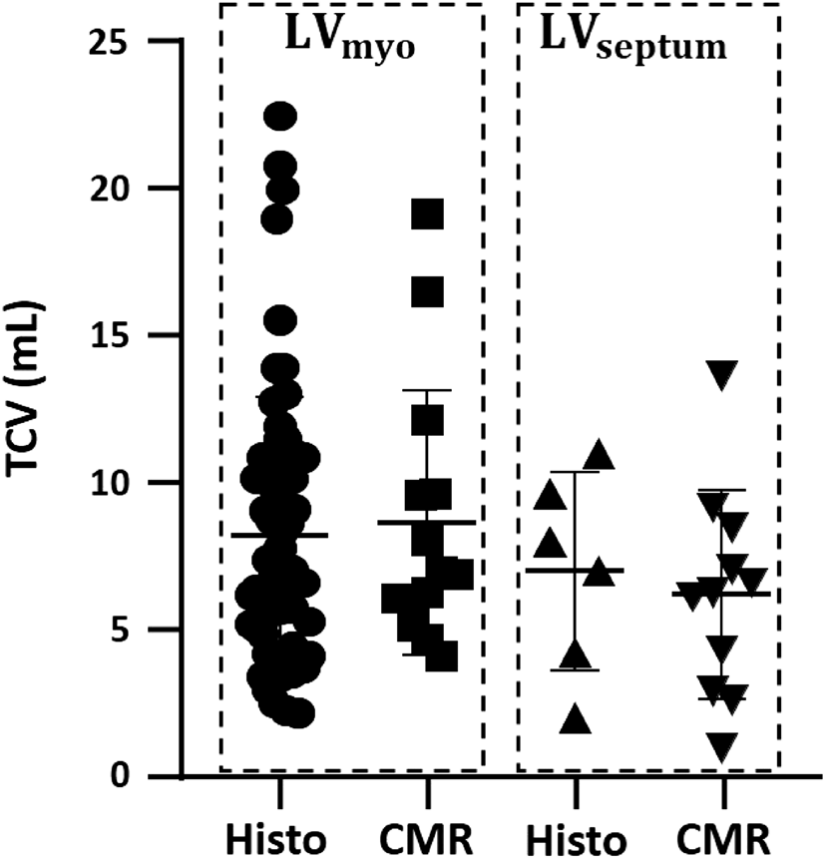
Box plots representing the comparison between total collagen volume (TCV) measured at T1 mapping against histology. Comparison between total collagen volume (TCV) measured in LV_myo_, LV_septum_ with histology (Histo) and T1 mapping (T1). The dashed boxes highlights the comparison between the two techniques. *LV*_*myo*_ left ventricular myocardium, *LV*_*septum*_ left ventricular septum. Data are presented as median and interquartiles values

**Fig. 5:**
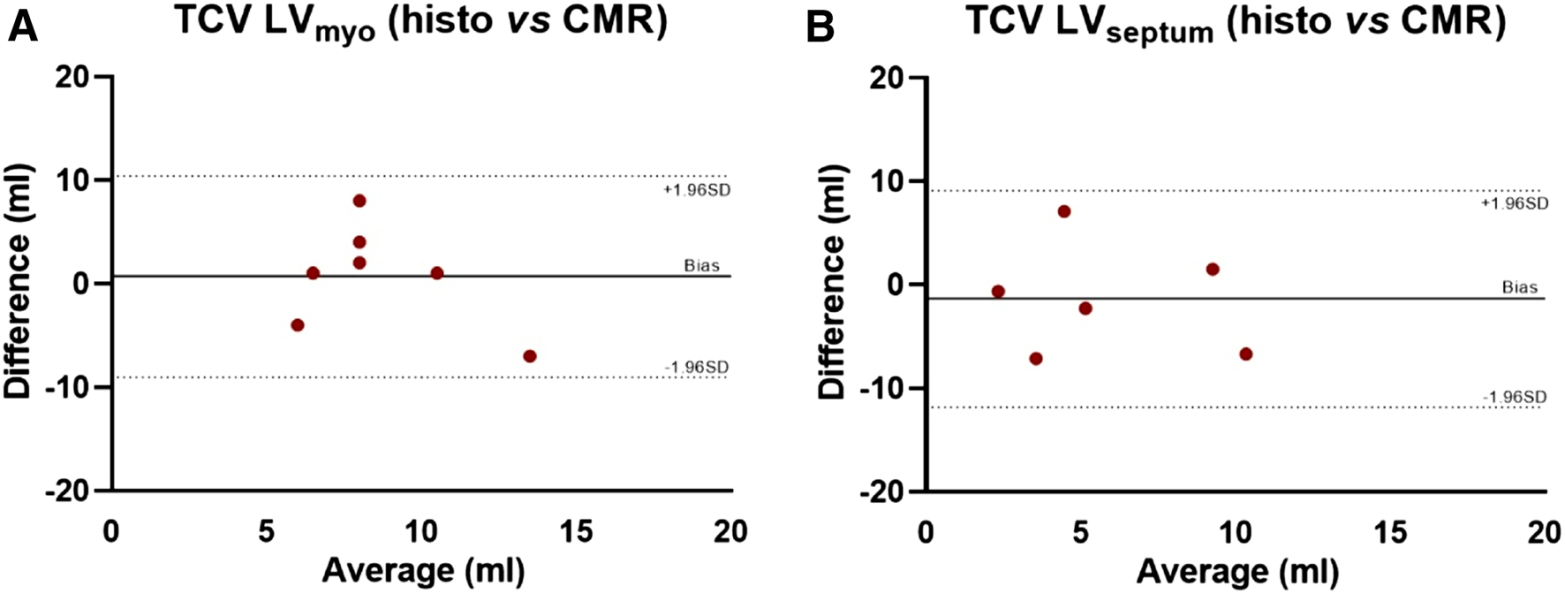
Bland–Altman plots between total collagen volume (TCV) measured in both histology and T1 mapping. **a** Bland–Altman plot comparing total collagen volume (TCV) measured by histology and T1 mapping at LV_myo_. **b** Same as A but for LV_septum_. The dashed lines indicate the upper and lower limits of agreement. The solid line indicates the difference between the means (Bias)

The MRI measured LV mass measured was 85 g ± 19 while the LV myocardial volume was 81 ml ± 18. No difference in TCV_CMR_ was observed between LV_myo_ and LV_septum_ measured with T1 mapping (Table 1). No difference in TCV_histo_ was observed between LV_myo_ and LV_septum_ measured with histology (Table 2). A comparison between the averaged TCV measured with T1 mapping and histology has shown to be not statistically different. A graphical representation of all the measurements is displayed in Fig. 4. No significant difference was observed between the TCV_CMR_ and TCV_histo_ in LV_myo_ and LV_septum_. We then performed a Bland–Altman analysis to evaluate the level of agreement between the TCV measured at histology against T1 mapping (Fig. 5). A moderate agreement was observed for TCV measured both in LV_myo_ (limits of agreement: − 9,  + 10, bias = 0.7, mean percentage difference = 8.2%) and LV_septum_ (limits of agreement: − 12, + 9, bias = 1.3, mean percentage difference = 8.4%).

**Table 2:** Histological collagen fraction parameters

Parameters	LV_myo_	LV_septum_	p-value
Basal collagen fraction (%)	12 ± 4	9 ± 4	0.200
Medial collagen fraction (%)	11 ± 2	9 ± 3	0.198
Apical collagen fraction (%)	12 ± 3	7.5	0.600
Total collagen fraction (%)	12 ± 3	9 ± 3	0.142
TCV_histo_ (ml)	8 ± 5	7 ± 3	0.565

Data above represent the histological collagen fraction parameters in LV_myo_, LV_septum_ and RV_endo_ at the basal, medial and apical levels

Data are presented as mean ± SD

*LV*_*myo*_ left ventricular myocardium, *LV*_*septum*_ left ventricular septum, *ECV* extracellular volume, *CVF* collagen volume fraction, *TCV* total collagen volume

## Discussion

Cardiovascular magnetic resonance (CMR) T1 mapping has emerged as a promising technique to detect myocardial scars, focal and diffuse fibrosis in different cardiomyopathies and even in patients with acute chest pain syndromes [6]. However, histological evaluation through RV biopsy still represents the gold standard for the quantification of myocardial fibrosis [16]. Nonetheless, biopsy always carries the risk of sampling error, perioperative adversities due to the intrinsic invasiveness of cardiac catheterizations and the exposure to ionizing radiations [8]. As reported by Schwartz et al. a considerable sampling error was evident when data obtained from two different myocardial biopsy were compared and that the error was the greatest for interstitial fibrosis quantification [9]. Moreover, the biopsied region represents only a small sample of the entire myocardium underestimating regional differences and collagen concentration [16]. For this reason, our study aimed to evaluate the collagen concentration through the measurement of total collagen volume (TCV) in the left ventricular myocardium (LV_myo_) and left ventricular septum (LV_septum_) with both T1 mapping and histological assessments in healthy Landrace pigs.

In our study, we have used volumetric shimming including a second order shim in a local volume around the heart, in order to keep the volumes of interest well within the region without banding artifacts. It has indeed been shown that significant errors in T1 may result already at relatively small off-resonance frequencies [17]. However, our estimation of the total collagen volume is based on ECV [13], and therefore on a comparison of T1 measurements with identical sequence parameters and shimming before- and after contrast administration. Therefore, it can be expected that, to some extent, variations of the apparent T1 due to large scale susceptibilities may level out in the ECV formalism.

To compare the TCV measured with histology against CMR T1 mapping different approaches have been used. Bull et al. [18] and Messroghli et al. [3] have previously shown a moderate correlation between both T1 native and ECV measurements against the collagen volume fraction (CVF) assessed histologically. However, the comparison between these measurements is flawed because the CVF measured at histology is expressed in percentage while the T1 native measurements are expressed in milliseconds. Moreover, both T1 native and late gadolinium enhancement (LGE) contrast measurements have different parameters that can influence the analysis. T1 native is influenced by the field strength and the pulse sequence used, the cardiac phase and region of measurement [17]. Different studies showed that T1 native is representative of edema more than of fibrosis given that an increase of tissue water and/or an increase of interstitial space are the most important determinants [2, 6]. On the other hand, LGE T1 values are dependent on contrast agent dosing, the time elapsing between administration and measurement and, lastly, renal clearance. This approach has value in the differential diagnosis of ischemic versus non-ischemic cardiomyopathy, but diffuse fibrosis could be undetected on LGE imaging because of the spatial resolution of LGE images and the absence of normal reference myocardium. The ECV is a marker of myocardial tissue remodeling and represents a more accurate estimation of fibrosis than both native and post-contrast T1 measurements [4, 6, 19, 20]. Nevertheless, ECV has been shown to overestimate collagen concentration since it takes both collagen and the interstitial space into account [16, 21].

In our study, a series of corrections were performed to provide a comparable absolute value expressed in millilitres of TCV for both T1 mapping and histology. In our analysis, TCV measured with T1 was extrapolated from the ECV corrected by LV_myo_ volume, which includes both the myocardium mass and density measured with MRI as already described in a previous study by our group [22]. Finally, to obtain the same unit of measure for histology, its CVF was corrected by the LV_mass_ measured with MRI. By then comparing the corrected TCV of both T1 mapping and histology no differences were found for both LV_myo_ and LV_septum_. Moreover, we investigated the accuracy of the TCV measured with both T1 mapping and histology with Bland–Altman analysis and found a similar level of agreement. Noteworthy, our results are in contrast from the ones published from the group of Ide et al., where they showed a difference between ECV and T1 measured in the septum versus the same parameters measured in the total myocardium [8]. This can be explained by the fact that compared to their study, we performed the same comparison in healthy hearts. Interestingly, Gottbrecht et al. performed a meta-analysis study in which they analyzed works where T1 mapping was conducted only in healthy adults with the goal to show the reproducibility and precision of the technique [23]. Their results are consistent with more precise measurements obtained for T1 than for ECV. Moreover, they also report a higher coefficient of variance for ECV which implies that a larger relative change in this parameter is required to detect differences between healthy adults. Even if we address healthy pigs, our results are in line with their observation. Our results confirm that the level of fibrosis may be heterogeneous in pathologic conditions and vary among segments. Although a T1 mapping evaluation of the whole heart should be encouraged compared to the septum only, the pathophysiology of the disease investigated, the quality of the images and the reproducibility of the measurements should always be considered.

In this study we were able to show that estimating TCV via T1 mapping could represent a promising technique for the assessment of collagen concentration in comparison to the respective histological measurements. From a clinical standpoint, this might be advantageous because it could avoid the perioperative complications of RV biopsies for a first assessment of myocardial fibrosis and collagen concentration. Moreover, it can be a useful non-invasive way to monitor disease progression and responsiveness to treatment in already chronic patients suffering from cardiomyopathies that would need anyway to undergo CMR control visits. Still, there are some missing points. More studies are needed to assess the sensitivity and specificity of T1 mapping in identifying TCV by comparing it with the histological analysis. Consequently, there is the need to find a physiological reference value of TCV in healthy adults to assess the normality of myocardial collagen concentration. These steps will allow to improve and build new algorithms for a better identification of collagen and fibrosis deposition through CMR.

Our study has several limitations. First of all, the animals were healthy, limiting the presence of potential differences within the ventricles concerning the collagen concentration or other inhomogeneities. The formula utilized for the calculation of CVF and developed by Flett et al. has not been validated with the MOLLI sequence. The myocardial density constant utilized has been validated only in humans and may be slightly different in swine. Moreover, the sample size used in our study is small and, thus, our results need to be confirmed with further studies. CMR suffers from a limited spatial resolution, thus partial volume averaging poses a challenge for ECV measures because other components of the ECV (i.e. blood and fat) can bias the evaluation. During imaging, care must be taken to avoid obliquity and ensure the myocardium is perpendicular to the plane of the image. Finally, HR is a known physiologic confounder of ECV mapping through MOLLI [24, 1].

## Conclusions

In this study, we showed the potential usefulness of TCV as a non-invasive parameter of collagen concentration for the LV. Further studies are needed to validate this parameter both in healthy and pathological models.

## Data Availability

Data are available upon request.

## Abbreviations

CMR: Cardiac magnetic resonance
CVF: Collagen volume fraction
ECV: Extracellular volume
LV: Left ventricle
LV_myo_: Left ventricular myocardium
LV_septum_: Left ventricular septum
MRI: Magnetic resonance imaging
RV: Right ventricle
T1 pre: Pre-contrast T1 relaxation time
T1 post: Post-contrast T1 relaxation time
TCV: Total collagen volume
TCV_CMR_: Total collagen volume by CMR
TCV_histo_: Total collagen volume by histology
